# Comparative Proteomic Study of Retinal Ganglion Cells Undergoing Various Types of Cellular Stressors

**DOI:** 10.1101/2023.10.06.561236

**Published:** 2023-10-11

**Authors:** Christopher R. Starr, Marina S. Gorbatyuk

**Affiliations:** Department of Optometry and Vision Science, University of Alabama at Birmingham, Birmingham, Alabama, USA, 35233

**Keywords:** Retina, Proteomics, Glaucoma, Excitotoxicity, Optic Neuropathy, Ophthalmology

## Abstract

Retinal ganglion cell (RGC) damage serves as a key indicator of various retinal degenerative diseases, including diabetic retinopathy (DR), glaucoma, retinal arterial and retinal vein occlusions, as well as inflammatory and traumatic optic neuropathies. Despite the growing body of data on the RGC proteomics associated with these conditions, there has been no dedicated study conducted to compare the molecular signaling pathways involved in the mechanism of neuronal cell death. Therefore, we launched the study using two different insults leading to RGC death: glutamate excitotoxicity and optic nerve crush (ONC). C57BL/6 mice were used for the study and underwent NMDA- and ONC-induced damages. Twenty-four hours after ONC and 1 hour after NMDA injection, we collected RGCs using CD90.2 coupled magnetic beads, prepared protein extracts, and employed LC-MS for the global proteomic analysis of RGCs. Statistically significant changes in proteins were analyzed using the Shiny Go program to identify GO biological processes and molecular functions resulting from the treatment. We identified unique and common alterations in protein profiles in RGCs undergoing different types of cellular stressors. Additionally, we observed the absence of certain proteins in treated RGCs compared to the control group. Our study not only identified both unique and shared proteomic changes but also laid the groundwork for the future development of a therapeutic platform for testing gene candidates for DR and glaucoma.

## 1 Introduction

Retinal ganglion cells (RGCs) are neurons located in the retina of the eye, playing a crucial role in vision by transmitting visual information from the retina to the brain. There are several types of RGCs, each with a specific function in processing visual information. They receive input from photoreceptor cells, rods and cones, and integrate this information before sending it to the brain via the optic nerve. The axons of RGCs form the optic nerve, which carries visual signals to the brain, where signals are further processed to create the visual perception. Once damaged, axons of the mammalian central nervous system (CNS) fail to regenerate. RGCs are of clinical relevance in diabetic retinopathy (Abu-El-Asrar et al., 2004), glaucoma (Joo et al., 1999), retinal arterial and retinal vein occlusions (Sucher et al., 1997) as well as optic neuropathies (Khan et al., 2021). In these diseases, RGCs may be irreversibly damaged. It is widely accepted that damaged RGCs not only serve as a model of optic neuropathies but also as the primary model for studying mechanisms of CNS axon degeneration and how therapies may promote regeneration.

After insult, both intrinsic and extrinsic factors contribute to whether CNS neurons survive, retain, or regenerate their axons. Although lowering intraocular pressure (IOP) is an approved treatment for glaucoma, it is not effective for every patient, and many other diseases affecting CNS neurons lack approved treatment options. In glaucoma, it has been reported that RGCs undergo apoptosis triggered by various factors, including autophagy, glutamate neurotoxicity, oxidative stress, neuroinflammation, immunity, and vasoconstriction (Kuehn et al., 2005; Shen et al., 2023). Autophagy can be induced by retinal hypoxia and axonal damage (Shen et al., 2023) while glutamate neurotoxicity is induced by the overstimulation of N-methyl-D-aspartate membrane receptors by glutamate, leading to progressive glaucomatous optic neuropathy (Shen et al., 2023).

In diabetes, retinal neuropathy involves progressive RGC death, axonal degeneration, and consequently, optic nerve degeneration (Bikbova et al., 2014). RGC loss occurs in diabetic patients even before the diagnosis of diabetic retinopathy. Furthermore, thinning of both the nerve fiber and the RGC layer has been documented in patients with diabetic retinopathy (Verma et al., 2009; Vujosevic and Midena, 2013; Verbraak, 2014) and animal diabetic models (Oleg S. Gorbatyuk, 2021; Pitale et al., 2021). One of the earliest experimental observations in animal models of diabetic retinopathy (DR) was the impairment of retrograde axonal transport in RGCs (Potilinski et al., 2020). Interestingly, this impairment was found to be even greater in type 1 diabetes than in type 2 diabetes, possibly due to metabolic dysfunctions contributing to optic nerve atrophy (Zhang et al., 1998). Under high glucose conditions, there was an observed increase in glutamate release, leading to significant extracellular glutamate accumulation and subsequent neurotoxicity of RGCs, further contributing to their deterioration(Ma et al., 2010).

Various approaches have been employed to analyze cellular signaling involved in RGC death and axonal deterioration. For instance, research groups have conducted unbiased proteomic screens of total mouse retina lysates (Magharious et al., 2011; Hollander et al., 2012; Kwong et al., 2021; Zhu et al., 2022) and used fluorescent-assisted cell sorting (FACS)(Belin et al., 2015) to isolate RGCs following optic nerve crush (ONC), one of the most extensively studied models of CNS axonal injury. However, most of these studies either examined whole retina lysates or assessed sorted RGCs at a time point (3 days after injury) when a significant amount of RGC apoptosis has already began (Goldenberg-Cohen et al., 2012). Another common injury model is N-methyl-D-aspartate (NMDA)-induced excitotoxicity, a model mimicking the glutamate-induced excitotoxicity associated with multiple neurodegenerative diseases such as amyotrophic lateral sclerosis (ALS), ischemic stroke, and traumatic brain injury (Guerriero et al., 2015; Ibanez et al., 2022; Rubino et al., 2023). To date, the only study examining proteomics in the context of NMDA-induced excitotoxicity of RGCs investigated proteomic changes in whole retinal lysates 12 hours after NMDA injection, which is a time point at which a substantial amount of RGC apoptosis is already in progress (Suo et al., 2022).

Understanding how CNS neurons respond to injury is crucial as we strive to develop therapeutic strategies for promoting neuroprotection, axon survival, and regeneration. Particularly, the significance of such studies lies in the development of neuroprotective interventions. Therefore, we initiated a comparative proteomic study, in which we analyzed the protein profiles of RGCs subjected to different cellular stress stimuli. The importance of our study lies in the fact that we not only identified differentially expressed proteins involved in two distinct cell death mechanisms but also revealed common biological processes that were similarly altered by different cell stress stimuli.

## 2 Results

### 2.1 Nontargeted quantitative proteomics of retinal ganglion cells.

**Figure 1-:**
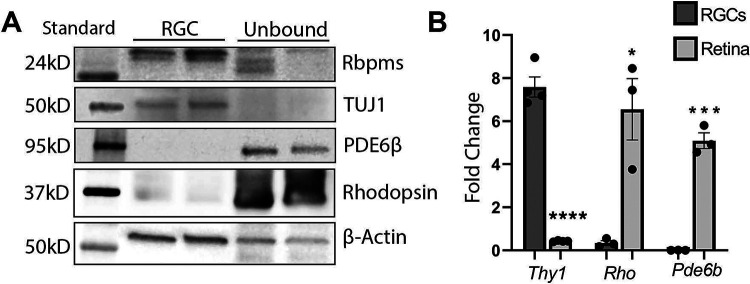
RGCs are enriched with Cd90.2 coupled magnetic beads. A) Western blot of select RGC and photoreceptor proteins indicating RGC enrichment. B) qRT-PCR analysis using primers against RGC or photoreceptor targets. * = p<0.05, *** = p<0.005, **** = p<0.001 (n=3-4). Data are shown as a standard deviation.

In our study, we adopted an approach to analyze molecular alterations in deteriorating RGCs before their cell death, even though the peaks of apoptosis occur at different time points. To investigate early proteomic changes in RGCs following ONC- or NMDA-induced injury, protein lysates from Thy1-magnetic bead-isolated RGCs underwent nano high-performance liquid chromatography/mass spectrometry (LC/MS). RGC enrichment was confirmed via western blot (WB) using antibodies against RGC proteins, RNA-binding protein with multiple splicing (Rbpms), Class III β-tubulin (Tubb3/TUJ1), and photoreceptor markers, phosphodiesterase 6β (Pde6β) and rhodopsin (Rho) (Fig. 1A). As expected, RGC markers Rbpms and Tubb3/TUJ1 were highly enriched in RGCs and notably reduced in cells not bound by Cd90.2-coupled magnetic beads, corresponding to the rest of the retinal cells (Fig. 1A). Furthermore, photoreceptor proteins Pde6β and rhodopsin were nearly undetectable in cells bound by the beads but highly expressed in unbound retinal cells. RGC enrichment was further validated by quantitative reverse transcriptase polymerase chain reaction (qRT-PCR) with primers against CD90/Thy1, Rho, and Pde6b (Fig. 1B). While Thy1 was significantly elevated in cells bound by the magnetic beads and reduced in unbound cells, *Rho* and *Pde6b* mRNA levels were high in unbound cells and significantly diminished in bound cells. Together, the WB and qRT-PCR results indicate reliable RGC enrichment was achieved in our study.

We analyzed identified proteins and prior to pathway analysis, grouped them according to the following parameters: 1) elevated in NMDA treated 2) reduced in NMDA treated 3) elevated in ONC treated 4) reduced in ONC 5) altered in NMDA and ONC.

### 2.2 NMDA Treatment shifts RGC metabolic signaling.

**Figure 2-:**
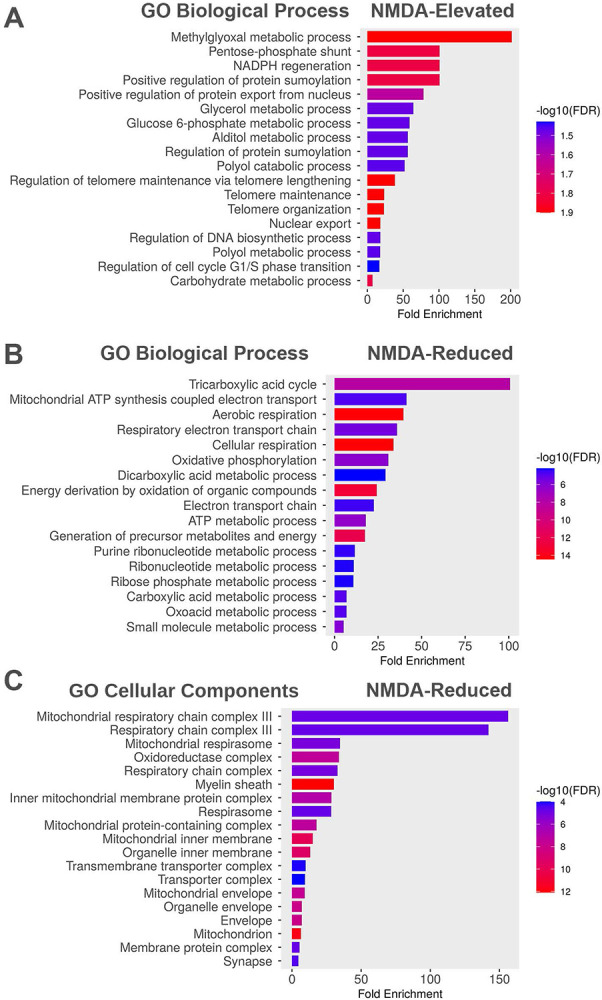
GO biological processes and cellular components of RGCs shift 1 hour after intravitreal NMDA injection. A) The most significantly elevated GO biological processes following NMDA-induced excitotoxicity. B) GO biological processes reduced by intravitreal NMDA injection. C) GO cellular components reduced by NMDA-induced excitotoxicity. False Discovery rate (FDR) ≥ 0.05.

To induce excitotoxic damage to the retina, we intravitreally injected 1 μl of 20 mM NMDA in PBS, following a protocol previously described (Guo et al., 2021). NMDA treatment resulted in significant alterations in multiple pathways, with a notable impact on metabolic processes (Fig. 2 and Table 1). NMDA treatment induced a substantial increase in methylglyoxal (MGO) signaling and the pentose-phosphate pathway (PPP), indicating the involvement of oxidative stress and a decrease in glutathione (GSH) levels, which is often associated with increased apoptosis (Nuamnaichati et al., 2020; Barisa et al., 2021) (Fig. 2A). Consistent with these findings, the levels of specific proteins related to these pathways, such as GLO1 (Lactoylglutathione lyase, Q9CPU0) in the MGO signaling pathway and 6-phosphogluconolactonase (Q9CQ60) in the PPP, were significantly elevated (Table 1). Additionally, NMDA treatment led to an increase in NADPH regeneration, a process known to be derived from Glucose 6-phosphate (G6P). This regeneration involves enzymes such as G6P dehydrogenase, which generates NADPH, and 6-phosphogluconolactonase (Q9CQ60), which converts 6-phosphogluconolactone to 6-phosphogluconate. The elevated presence of 6-phosphogluconolactonase (Q9CQ60) in NMDA-treated RGCs was observed (Table 1). Furthermore, our study revealed an enhancement in protein sumoylation in NMDA-treated RGCs. This posttranslational modification plays a pivotal role in various biological functions, including nuclear-cytosolic transport, transcriptional regulation, apoptosis, and protein stability, among others.

Therefore, these findings demonstrate that NMDA treatment has a significant impact on metabolic signaling pathways in retinal ganglion cells, suggesting a link to oxidative stress, altered GSH levels, and increased apoptosis. Moreover, the study highlights the involvement of NADPH regeneration and protein sumoylation as part of the cellular responses to NMDA-induced excitotoxicity.

We then plotted proteins diminished following NMDA injections using the ShinyGo program. Fig. 2B and Table 1 clearly demonstrate that NMDA treatment results in a reduction of proteins associated with critical cellular processes, including the tricarboxylic acid (TCA) cycle, mitochondrial ATP-coupled electron transport, aerobic respiration, and the respiratory electron transport chain. This reduction is particularly noteworthy as it predominantly affects mitochondrial proteins involved in essential functions (Table 1), ultimately leading to a decrease in adenosine triphosphate (ATP) production. Notably, NMDA excitotoxicity predominantly affects proteins crucial for ATP generation, underscoring its impact on cellular energy metabolism. Furthermore, our study identified specific cellular components that were enriched as a result of NMDA treatment. This enrichment notably included the mitochondrial respiratory chain complex III (involved in oxidative phosphorylation) and the oxidoreductase complex (specifically complex I: NADH-ubiquinone oxidoreductase). This elevation in these components aligns with the observed increase in various metabolic cellular processes (as depicted in Fig. 2C).

Collectively, our findings indicate that NMDA excitotoxicity in retinal ganglion cells leads to rapid perturbations in metabolic processes. These perturbations are characterized by a reduction in ATP production pathways and a simultaneous shift of glycolysis towards the PPP. This shift reflects the intricate interplay between NMDA-induced damage and cellular metabolic responses, shedding light on the complex mechanisms involved in excitotoxicity-induced cellular alterations.

### 2.3 ONC Results in an Early Shift in DNA Repair and Telomere Maintenance in RGCs.

**Figure 3 -:**
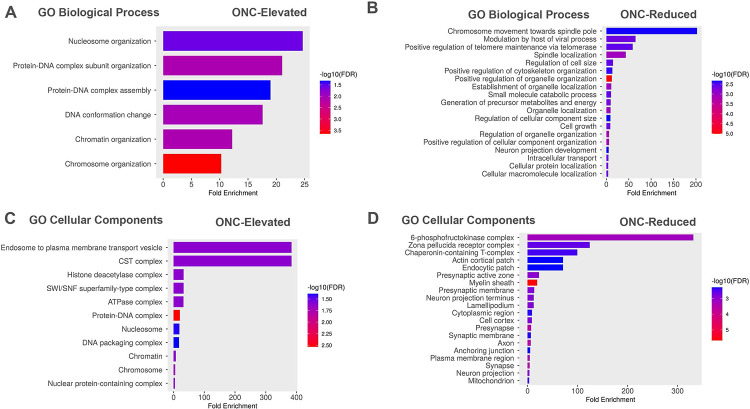
GO biological processes and cellular components of RGCs shift 24 hours after ONC. A) The most significantly elevated GO biological processes following traumatic injury to the optic nerve. B) GO biological processes reduced by ONC. C) GO cellular components elevated by ONC. D) GO cellular components reduced 24 hours after ONC. False Discovery rate (FDR) ≥ 0.05.

RGCs were isolated 24 hours after the ONC procedure and subjected to LC/MS analysis. Examples of biological processes elevated following ONC include nucleosome organization, formation of protein-DNA complexes, DNA chromatin changes, and chromatin organization (Fig. 3A). These biological processes were accompanied by an increase in chromodomain-helicase-DNA-binding protein 4 (Q6PDQ2), histone H2B type 3-A (Q9D2U9), Histone H2B type 1-H (Q64478), and other nuclear proteins (Table 2). Among the reduced biological processes following ONC, we found that positive regulation of telomer and telomerase and spindle localization includes organelle localization (Fig. 3B and Table 2).

The GO Cellular component analysis demonstrates that an increase in proteins in ONC-processed RGCs was associated with endosome complex formation and CST, a cellular multiprotein complex involved in telomere maintenance (Fig. 3C). Therefore, it is not surprising that the endosomal Ras-related protein Rab-18 (P352930) was elevated. Other examples are the CST complex subunit STN1 (Q8K2X3) and High Mobility Group protein B3 (O54879) regulating the mechanisms of DNA replication, transcription, recombination, and repair that were significantly elevated in the ONC-processed RGCs (Table 2). We then found that reduced proteins were responsible for the GO cellular components, including the 6-phosphofructokinase complex and chaperon-containing T complex (CCT) (Fig. 3D). These findings were in accordance with the expression levels of individual proteins such as ATP-dependent 6-phosphofructokinases (P12382 and P47857) and T-complex protein 1 subunit gamma (TRiC or P80318). Overall, these data suggested that ONC causes chromatin and DNA reorganization in RGCs and reduces the cellular ability to repair DNA damage.

### 2.4 Proteins altered following both NMDA-induced excitotoxicity and ONC.

**Figure 4 –:**
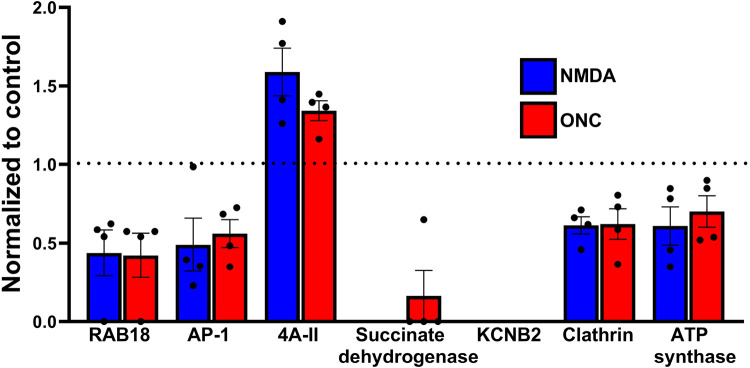
Select proteins with signficantly altered levels after both NMDA injection and ONC. Levels depicted have been normalized to control. All proteins that are changed in both models of RGC injury are listed in table 3 (n=4). Data are shown as a standard error.

One of the primary objectives of this study was to identify proteins that exhibit changes in multiple models of neuronal injury. In pursuit of this goal, we successfully identified proteins that exhibited alterations in both models (Fig. 4 and Table 3). Remarkably, the majority of these protein level changes, whether upregulation or downregulation, were notably consistent across both groups. Specifically, we observed significant upregulation of eukaryotic initiation factor 4A-II (4A-II), small nuclear ribonucleoprotein D3 (SNRD3), and STN1, alongside significant downregulation of Ras-related protein (Rab-18), AP-1, Clathrin, ATP synthase subunit D, and sodium and chloride-dependent GABA transporter 3 in both treated RGCs when compared to the control group. Fig. 4, which provides a visual representation of these modified protein levels, also highlights the unique absence of succinate semialdehyde dehydrogenase (SSADH) and KCNB2 (potassium voltage-gated channel subfamily B member 2) in both RGC treatments as compared to control.

The analysis of differentially expressed proteins revealed 16 proteins with significantly reduced expression, four proteins with significantly increased expression, one protein exhibiting an opposite response pattern to the stimulus in both types of treated RGCs, and two proteins uniquely present in the control group.

## 3 Discussion

In the present study, we examined acute changes in RGC global protein expression following NMDA injection or ONC. We provide evidence that NMDA treatment alters multiple signaling pathways, many of which are metabolic. In addition, we identified several biological processes impacted by ONC. We identified common proteins manifesting a decline and incline in expression and unique proteins absent in RGC undergoing both types of injury.

Classically, the primary cause of glutamate excitotoxicity is ATP depletion and impaired glutamate transport, resulting in a buildup of extracellular glutamate, leading to excitotoxicity by overloading NMDA receptor (NMDAR)-expressing cells with Na+ and Ca2+. Glutamate excitotoxicity is known to be involved in various diseases, including Alzheimer's (Tannenberg et al., 2004), Parkinson's (Verma et al., 2022), or Huntington's (Girling et al., 2018), and predicted to be involved in choroidal vessel occlusion, glaucoma, and diabetic retinopathy (Lucas and Newhouse, 1957; Massey and Miller, 1987, 1990; Thoreson and Witkovsky, 1999; Yumnamcha et al., 2020). Elevated Ca2+ can lead to changes in several Ca2+-sensitive signaling cascades and eventually mitochondrial-mediated apoptosis (Verma et al., 2022). An increase in intracellular Na+ can lead to cell swelling. NMDA-mediated excitotoxicity leads to synaptic degeneration and dendritic pruning before cell death. By isolating RGCs one hour after NMDA injection, we hoped to see how the RGCs are altered in the time window before full-fledged apoptosis begins. We did indeed observe perturbations in several biological processes after NMDA injection. We report that early NMDA-induced excitotoxicity leads to a substantial elevation of MGO and the PPP, indicating a potential involvement of oxidative stress and a reduction in the levels of GSH, which is often associated with apoptosis. Despite this, we did not observe a significant change in GO biological processes pertaining to apoptosis or any other known form of cell death, indicating we did indeed perform proteomic analysis at a time point after NMDA-induced excitotoxicity where apoptotic signaling is not yet elevated. In addition, we also discovered that many of the cellular processes upregulated by excitotoxicity involve changes in the nucleus, such as nuclear protein export, and regulation of DNA processes (Fig. 2A). These specific changes could represent heterogeneous nuclear ribonucleoprotein A1, A1/B1, small nuclear ribonucleoprotein D3.

We also show that NMDA treatment reduces many GO biological processes. Of note, most proteins reduced by intravitreal NMDA injection are involved in ATP production. It is possible that the calcium elevation that occurs during excitotoxicity could be the major driving force behind excitotoxic cell death. Cells respond to this is by sequestering this excess calcium in the endoplasmic reticulum and mitochondria. Mitochondria is the site of oxidative phosphorylation, a process that generates ATP and is regulated by calcium (Glancy et al., 2013). This is consistent with our findings on a reduction of GO biological processes pertaining to ATP production and a decline in GO cellular components pertaining to various mitochondrial complexes.

The murine optic nerve crush injury model is the most commonly used model of RGC injury that mimics molecular events occurring upon traumatic optic neuropathy, glaucoma, etc. (Tang et al., 2011) Moreover, the prevalence of optic neuropathy in diabetics could increase with diabetes duration (Hua et al., 2019) In this model, the crush injury to the optic nerve leads to retinal ganglion cell apoptosis. This disease model can be used to study the general processes and mechanisms of neuronal death and survival, which is essential for the development of therapeutic measures. In addition, pharmacological and molecular approaches can be used in this model to identify and test potential therapeutic reagents to treat different types of optic neuropathy. Interventions that are neuroprotective for RGCs challenged with ONC are usually neuroprotective for other CNS neurons (Le Pichon et al., 2017). In addition, axon regeneration promoting treatments discovered with the ONC model can typically promote axon regeneration in the spinal cord (Bhowmick and Abdul-Muneer, 2021). Following ONC, axonal components briefly travel retrogradely prior to the axons returning to the site of injury, only to fail to pass the crush site. In fact, knocking out an inhibitor of the retrograde injury response of RGCs, dual-leucine zipper kinase (DLK), leads to robust RGC survival (Watkins et al., 2013), indicating that retrograde signaling is crucial for cell death signaling following ONC. Based on this, in the earliest response to ONC, we would anticipate changes in the machinery responsible for transport and localization. As expected, we did identify reductions in GO biological processes pertaining to the localization of organelles cellular proteins and cellular macromolecules. After injuring the axon with ONC, we expected to observe changes in synaptic GO cellular components. To that end, we did detected changes in components of synaptic membrane, synapse, neuronal projections, the presynapse, and the presynaptic membrane region. These changes are presented by the decline in multiple proteins including Catenin beta-1, Sodium- and chloride-dependent GABA transporter 3, Synaptogyrin-3 and Synaptotagmin-1. Together, these changes in GO biological processes and cellular components after ONC suggest that, as expected, damaging the axon not only disrupts axonal components but also the cellular localization of various cellular entities.

In both RGC degenerative models, we identified common proteins that responded to cellular insults in a similar manner, as well as proteins that exhibited a unique decline in expression in both challenged RGCs. While our current study has its limitations, future investigations should prioritize the validation of the roles of these proteins in RGC survival. In particular, it would be interesting to assess the role of these proteins in RGC survival during the development of diabetic retinopathy or glaucoma. For example, Rab18 deficiency detected in both types of challenged RGCs is the molecular deficit underlying Warburg micro syndrome, characterized by eye, nervous system, and endocrine abnormalities.(Handley and Sheridan, 1993) Moreover, a recent study highlighted the involvement of Rab18 in lipid metabolism in human diabetic adipose tissue and demonstrated that Rab-18 contributes to insulin resistance in obese individuals. (Pulido et al., 2011; Guzman-Ruiz et al., 2020) Another example is DnaJC8 protein, the effect of which is closely associated with the aggregation of polyQ-containing proteins in a cellular model of spinocerebellar ataxia type 3 (SCA3).(Ito et al., 2016) The authors have shown that DnaJC8 overexpression significantly reduces polyQ aggregation and apoptosis. Therefore, DnaJC8 should be validated for its neuroprotective role in the survival of RGCs undergoing both cellular stressors. Finally, both KCNB2 and SSADH are exclusively present in control C57BL6 RGCs and are absent in both types of challenged RGCs. Similar to SSADH deficiency representing a genetic disorder resulting from the aberrant metabolism of GABA (Didiasova et al., 2020), loss of SSDAH could be a consequence of a defective GABA catabolism in both types of stressed RGCs.

In summary, our study was designed to not only identify both individual and shared proteomic changes in retinal ganglion cells undergoing different stress stimuli just before initiating a pro-apoptotic cell death program but also to lay the groundwork for the future development of a therapeutic platform for testing gene candidates contributing to retinal diseases such as diabetic retinopathy and glaucoma.

## 4 Conclusion

Here we report that challenging RGCs alters the levels of various proteins and therefore likely impact cellular signaling at the onset of damage. We utilized unbiased, non-targeted proteomics to identify proteins and GO biological processes altered after ONC or NMDA-induced excitotoxicity. NMDA-induced excitotoxicity resulted in altered metabolic signaling. We observed a noticeable reduction in proteins and GO biological processes involved in ATP production. NMDA led to an increase in MGO signaling and the PPP, indicating the involvement of oxidative stress and a decrease in GSH levels. RGCs experienced a shift in DNA repair and telomere maintenance 24 hours after ONC. Further characterization of these altered proteins and signaling pathways may be vital for the creation of therapeutic countermeasures for neurodegenerative diseases.

## 5 Methods

### 5.1 Animals

All animal procedures were approved by The University of Alabama at Birmingham institutional animal use and care (IACUC) committee and in accordance with the statement for the Use of Animals in Ophthalmic and Vision Research by The Association for Research in Vision and Ophthalmology (ARVO). C57BL/6J mice were purchased from Jackson Laboratory (Bar Harbor, ME). An equal number of female and male mice were used in this study. For all procedures, mice were anesthetized with ketamine (100 mg/kg) and xylazine (10mg/kg).

To induce injury to RGCs, mice were either intravitreally injected with 1μl of 20mM N-Methyl D aspartic acid (NMDA) or underwent optic nerve crush (ONC) surgery as previously described (Guo et al., 2021). Briefly, for ONC, fine tweezers (Dumont#5; Fine Science Tools (FST) Item No. 11254-20 or Dumont#55; FST Item No. 11255-20) were used to create a small incision in the conjunctiva and then maneuvered between extraocular muscles to access the optic nerve, which was gently squeezed for 5 seconds at a location approximately 1mm posterior to the globe using Dumont #5 tweezers.

### 5.2 RGC Isolation

To isolate RGCs, we used a method that has previously been verified with slight modifications. Briefly, animals from each group were euthanized with CO_2_ asphyxiation and their retinas were harvested in cold neurobasal medium and placed in a 37°C water bath for 5 minutes. The neurobasal media was removed and replaced with fresh pre-warmed neurobasal media containing papain (0.06mg/ml or 33.4 U/mg) and 5mM L-cysteine and incubated 20 minutes at 37°C. The papain solution was removed and replaced with neurobasal containing 2mM L-Glutamine (Gibco; Catalog no. 25030081) B-27 supplement (Gibco; Catalog no. 17504044) and 10% FBS (Gibco; Catalog no. 26140079). The retinas were dissociated by gently pipetting up and down with a wide bore 1ml pipette tip. Cells were then centrifuged at 450g for 8 minutes and resuspended in 90 μl of isolation buffer (DPBS + 0.5% BSA + 2mM EDTA) containing 25 μg/ml DNAse I + 5mM MgCl2. Cells were filtered through a 30μm cell strainer and incubated with CD90.2 magnetic beads (Miltenyi Biotec; Catalog no. 130-121-278) at 4°C for 10 minutes and then isolated using MACs LS (Miltenyi Biotec; Catalog no. 130-042-401) columns following the manufacturer’s instructions. After isolation, cells were washed with DPBS, centrifuged at 450g for 8 minutes and their pellets stored at −70°C prior to proteomics sample preparation and LC/MS analysis.

### 5.3 Proteomics

#### 5.3.1 LC/MS

Proteomics analysis was carried out as previously described with minor changes (Ludwig et al., 2016), within section 2.5 nLC-ESI-MS2 under Protein IDs for GeLC. Proteins from isolated RGCs were extracted using T-PER^™^ Mammalian Protein Extraction Reagent (Thermo Fisher Scientific, Cat.# 78510) supplemented with HALT protease inhibitor cocktail (Thermo Fisher Scientific, Cat.# 78425), and benzonase nuclease (Sigma, E1014) following manufacturers instructions. Lysates were quantified using Pierce BCA Protein Assay Kit (Thermo Fisher Scientific, Cat.# 23227). Samples were prepared in NuPAGE LDS sample buffer (1x final conc., Invitrogen, Cat.# NP0007) and reduced with DTT then denatured at 70°C for 10min prior to loading 20μg onto Novex NuPAGE 10% Bis-Tris Protein gels (Invitrogen, Cat.# NP0315BOX) and separated appropriately (@ 200 constant V). The gels were stained overnight with Novex Colloidal Blue Staining kit (Invitrogen, Cat.# LC6025). Following de-staining, each entire lane was cut into multiple MW and equilibrated in 100 mM ammonium bicarbonate (AmBc), each gel plug was then digested overnight with Trypsin Gold, Mass Spectrometry Grade (Promega, Cat.# V5280) following manufacturer’s instruction. Peptide extracts were reconstituted in 0.1% Formic Acid/ddH2O at 0.1μg/μL.

Peptide digests (8μL each) were injected onto a 1260 Infinity nHPLC stack (Agilent Technologies), and separated using a 75 micron I.D. x 15 cm pulled tip C-18 column (Jupiter C-18 300 Å, 5 micron, Phenomenex). This system runs in-line with a Thermo Q Exactive HFx mass spectrometer, equipped with a Nanospray Flex^™^ ion source (Thermo Fisher Scientific), and all data were collected in CID mode. The nHPLC is configured with binary mobile phases that includes solvent A (0.1%FA in ddH2O), and solvent B (0.1%FA in 15% ddH2O/85% ACN), programmed as follows; 10min @ 5%B (2μL/min, load), 30min @ 5%-40%B (linear: 0.5nL/min, analyze), 5min @ 70%B (2μL/min, wash), 10min @ 0%B (2μL/min, equilibrate). Following each parent ion scan (300-1200m/z @ 60k resolution), fragmentation data (MS2) were collected on the top most intense 18 ions @7.5K resolution. For data dependent scans, charge state screening and dynamic exclusion were enabled with a repeat count of 2, repeat duration of 30s, and exclusion duration of 90s.

MS data conversion and searches The XCalibur RAW files were collected in profile mode, centroided and converted to MzXML using ReAdW v. 3.5.1. The mgf files were created using MzXML2Search (included in TPP v. 3.5) for all scans. The data was searched using SEQUEST (Thermo Fisher Scientific), which is set for three maximum missed cleavages, a precursor mass window of 20ppm, trypsin digestion, variable modification C @ 57.0293, and M @ 15.9949 as a base setting. Searches were performed with the mus musculus species specific subset of the UniProtKB database.

#### 5.3.2 Peptide filtering, grouping, and quantification

The list of peptide IDs generated based on SEQUEST search results were filtered using Scaffold (Protein Sciences, Portland Oregon). Scaffold filters and groups all peptides to generate and retain only high confidence IDs while also generating normalized spectral counts (N-SC’s) across all samples for the purpose of relative quantification. The filter cut-off values were set with minimum peptide length of >5 AA’s, with no MH+1 charge states, with peptide probabilities of >80% C.I., and with the number of peptides per protein ≥2. The protein probabilities will be set to a >99.0% C.I., and an FDR<1.0. Scaffold incorporates the two most common methods for statistical validation of large proteome datasets, the false discovery rate (FDR) and protein probability (Keller, Nesvizhskii, Weatherly). Relative quantification across experiments were then performed via spectral counting (Old, Liu), and when relevant, spectral count abundances will then be normalized between samples (Hyde).

#### 5.3.3 Statistical analysis

For generated proteomic data, two separate non-parametric-like statistical analyses were performed between each pair-wise comparison. These analyses included; A) the calculation of weight values by significance analysis of microarray (SAM; cut off >∣0.8∣ combined with, B) T-Test (single tail, unequal variance, cut off of p < 0.05), which are then sorted according to the highest statistical relevance in each comparison. For SAM, the weight value (W) is a function statistically derived that approaches significance as the distance between the means (μ1-μ2) for each group increases, and the SD (δ1-δ2) decreases using the formula: W=(μ1-μ2)/(δ1-δ2). For protein abundance ratios determined with N-SC’s, we set a 1.5-fold change as the threshold for significance, determined empirically by analyzing the inner-quartile data from the control experiments using ln-ln plots, where the Pierson’s correlation coefficient (R) is 0.98, and >99% of the normalized intensities fell between the set fold change. Each of the tests (SAM, Ttest, and fold change) must be passed for to be considered significant.

#### 5.3.4 Systems Analysis

Gene ontology assignments and pathway analysis were carried out using ShinyGO (Ge et al., 2020).

## Figures and Tables

**Table 1-:** Proteins differentially expressed following NMDA treatment

Protein Name	Accession Number	p-value	Level compared to control
40S ribosomal protein S10	P63325	0.011	elevated
Peptidyl-prolyl cis-trans isomerase FKBP1A	P26883	0.030	elevated
SUMO-activating enzyme subunit 1	Q9R1T2	0.001	elevated
Probable ATP-dependent RNA helicase DDX28	Q9CWT6	0.029	elevated
Metastasis-associated protein MTA2	Q9R190	0.011	elevated
6-phosphogluconolactonase	Q9CQ60	0.014	elevated
Plasminogen activator inhibitor 1 RNA-binding protein	Q9CY58	0.043	elevated
Phosducin	Q9QW08	0.020	elevated
Eukaryotic translation initiation factor 4B	Q8BGD9	0.015	elevated
DnaJ homolog subfamily C member 8	Q6NZB0	0.041	elevated
ADP-ribosylation factor-like protein 3	Q9WUL7	0.036	elevated
Hypoxanthine-guanine phosphoribosyltransferase	P00493	0.048	elevated
Inositol (Myo)-1(Or 4)-monophosphatase 1	Q924B0	0.033	elevated
DNA-(apurinic or apyrimidinic site) lyase	P28352	0.001	elevated
Ras GTPase-activating protein 4	Q6PFQ7	0.032	elevated
Lactoylglutathione lyase	Q9CPU0	0.027	elevated
Cold-inducible RNA-binding protein	P60824	0.034	elevated
Eukaryotic initiation factor 4A-II	E9Q561	0.011	elevated
Guanylate kinase	Q64520	0.042	elevated
Elongation factor 1-beta	O70251	0.039	elevated
Heterogeneous nuclear ribonucleoprotein F	Q9Z2X1	0.018	elevated
T-complex protein 1 subunit delta	P80315	0.025	elevated
14-3-3 protein epsilon	D6REF3	0.019	elevated
SUMO-activating enzyme subunit 2	Q9Z1F9	0.041	elevated
Acidic leucine-rich nuclear phosphoprotein 32 family member B	Q9EST5	0.049	elevated
Triosephosphate isomerase	P17751	0.007	elevated
Aspartate aminotransferase, cytoplasmic	P05201	0.050	elevated
Tubulin alpha-1C chain	P68373	0.035	elevated
Phosphoglycerate mutase 1	Q9DBJ1	0.029	elevated
Heterogeneous nuclear ribonucleoprotein A1	P49312	0.027	elevated
Heterogeneous nuclear ribonucleoproteins A2/B1	O88569	0.043	elevated
Small nuclear ribonucleoprotein D3	Q91VM2	0.031	elevated
CST complex subunit STN1	Q8K2X3	0.049	elevated
UMP-CMP kinase	Q9DBP5	0.043	reduced
ATP-dependent 6-phosphofructokinase, platelet type	Q9WUA3	0.035	reduced
NADH-ubiquinone oxidoreductase 75 kDa subunit, mitochondrial	Q91VD9	0.016	reduced
Aconitate hydratase, mitochondrial	Q99KI0	0.016	reduced
Inorganic pyrophosphatase	Q9D819	0.038	reduced
Sodium/potassium-transporting ATPase subunit alpha-3	Q6PIC6	0.020	reduced
Sodium/potassium-transporting ATPase subunit alpha-1	Q8VDN2	0.022	reduced
N(G),N(G)-dimethylarginine dimethylaminohydrolase 1	Q9CWS0	0.044	reduced
Voltage-dependent anion-selective channel protein 2	Q60930	0.029	reduced
Protein piccolo	Q9QYX7	0.021	reduced
Cytochrome b-c1 complex subunit 2, mitochondrial	Q9DB77	0.017	reduced
Clathrin coat assembly protein AP180	Q61548	0.021	reduced
Mitochondrial carrier homolog 2	Q791V5	0.022	reduced
ATP synthase subunit d, mitochondrial	Q9DCX2	0.020	reduced
Leucine-rich repeat-containing protein 59	Q922Q8	0.013	reduced
Dihydrolipoyllysine-residue acetyltransferase component of pyruvate dehydrogenase complex, mitochondrial	Q8BMF4	0.044	reduced
Synaptogyrin-3	Q8R191	0.032	reduced
Enoyl-CoA delta isomerase 1, mitochondrial	P42125	0.049	reduced
MICOS complex subunit Mic60	Q8CAQ8	0.031	reduced
Ras-related protein Rab-18	P35293	0.002	reduced
Protein NipSnap homolog 1	O55125	0.037	reduced
Fumarate hydratase, mitochondrial	P97807	0.009	reduced
Transportin-1	Q8BFY9	0.043	reduced
MAGUK p55 subfamily member 2	Q9WV34	0.050	reduced
Calcium-binding mitochondrial carrier protein Aralar1	Q8BH59	0.001	reduced
Cytochrome c1, heme protein, mitochondrial	Q9D0M3	0.025	reduced
Alpha-actinin-4	P57780	0.018	reduced
Succinyl-CoA ligase [ADP-forming] subunit beta, mitochondrial	Q9Z2I9	0.025	reduced
Transforming protein RhoA	Q9QUI0	0.019	reduced
Microtubule-associated protein 6	Q7TSJ2	0.010	reduced
Spna2 protein	B9EKJ1	0.030	reduced
Isocitrate dehydrogenase [NAD] subunit, mitochondrial	Q91VA7	0.048	reduced
AP-1 complex subunit beta-1	O35643	0.024	reduced
Carbonic anhydrase 14	Q9WVT6	0.012	reduced
40S ribosomal protein S3a	P97351	0.023	reduced
Pyruvate dehydrogenase E1 component subunit beta, mitochondrial	Q9D051	0.023	reduced
60S acidic ribosomal protein P0	P14869	0.014	reduced
Ornithine aminotransferase, mitochondrial	P29758	0.047	reduced
Sideroflexin-3	Q91V61	0.003	reduced
Sodium- and chloride-dependent GABA transporter 3	P31650	0.044	reduced
NADH dehydrogenase [ubiquinone] iron-sulfur protein 8, mitochondrial	Q8K3J1	0.007	reduced
Mannose-P-dolichol utilization defect 1 protein	Q9R0Q9	0.003	reduced
Catenin (Cadherin associated protein), alpha 1	Q6NV50	0.041	reduced
Importin subunit beta-1	P70168	0.046	reduced
Catenin beta-1	Q02248	0.010	reduced
Cytochrome b-c1 complex subunit Rieske, mitochondrial	Q9CR68	0.020	reduced
Pyridoxal kinase	Q8K183	0.034	reduced

**Table 2-:** Proteins Differentially expressed following ONC

Protein Names	Accession Number	p-value Control vs ONC	Level compared to control
Splicing factor 3A subunit 1	Q8K4Z5	0.050	elevated
CST complex subunit STN1	Q8K2X3	0.028	elevated
Stomatin-like protein 2, mitochondrial	Q99JB2	0.015	elevated
DnaJ homolog subfamily C member 8	Q6NZB0	0.047	elevated
Hepatoma-derived growth factor-related protein 2	Q3UMU9	0.013	elevated
Ddx3x protein	B9EKE9	0.017	elevated
von Willebrand factor A domain-containing protein 8	Q8CC88	0.039	elevated
High mobility group protein B3	O54879	0.003	elevated
DNA-(apurinic or apyrimidinic site) lyase	P28352	0.004	elevated
Inosine-5'-monophosphate dehydrogenase 1	P50096	0.047	elevated
Peptidyl-prolyl cis-trans isomerase A	P17742	0.020	elevated
Histone H2B type 3-A	Q9D2U9	0.024	elevated
Acidic leucine-rich nuclear phosphoprotein 32 family member E	P97822	0.035	elevated
Histone H2B type 1-H	Q64478	0.024	elevated
Cullin-associated NEDD8-dissociated protein 1	Q6ZQ38	0.048	elevated
Heterogeneous nuclear ribonucleoprotein F	Q9Z2X1	0.029	elevated
Eukaryotic initiation factor 4A-II	E9Q561	0.012	elevated
Protein DEK	Q7TNV0	0.030	elevated
Ras-related protein Rab-6A	P35279	0.028	elevated
Peroxiredoxin-1	P35700	0.042	reduced
Transketolase	P40142	0.028	reduced
Dihydropyrimidinase-related protein 2	O08553	0.007	reduced
Small nuclear ribonucleoprotein D3	Q91VM2	0.013	reduced
ATP synthase subunit d, mitochondrial	Q9DCX2	0.028	reduced
Cytoplasmic dynein 1 heavy chain 1	Q9JHU4	0.045	reduced
T-complex protein 1 subunit gamma	P80318	0.037	reduced
Fumarate hydratase, mitochondrial	P97807	0.038	reduced
Actin-related protein 3	Q99JY9	0.022	reduced
Synaptotagmin-1	P46096	0.028	reduced
ATP-dependent 6-phosphofructokinase, liver type	P12382	0.008	reduced
T-complex protein 1 subunit alpha	P11983	0.021	reduced
Protein kinase, cAMP dependent regulatory, type II alpha	Q8K1M3	0.039	reduced
Clathrin coat assembly protein AP180	Q61548	0.026	reduced
Carbonic anhydrase 14	Q9WVT6	0.045	reduced
Cofilin-1	P18760	0.024	reduced
Mitochondrial 2-oxoglutarate/malate carrier protein	Q9CR62	0.048	reduced
Ras-related protein Rab-18	P35293	0.002	reduced
Kras protein	Q5J7N1	0.027	reduced
AP-1 complex subunit beta-1	O35643	0.005	reduced
Enoyl-CoA hydratase, mitochondrial	Q8BH95	0.045	reduced
Transforming protein RhoA	Q9QUI0	0.037	reduced
Prohibitin	P67778	0.044	reduced
Transportin-1	Q8BFY9	0.040	reduced
26S proteasome non-ATPase regulatory subunit 2	Q8VDM4	0.023	reduced
Tubby-related protein 1	Q9Z273	0.042	reduced
Pyridoxal phosphate phosphatase	P60487	0.031	reduced
Protein NipSnap homolog 2	O55126	0.019	reduced
ATP-dependent 6-phosphofructokinase, muscle type	P47857	0.045	reduced
RNA-binding protein EWS	Q61545	0.018	reduced
Glycogen phosphorylase, brain form	Q8CI94	0.004	reduced
Ornithine aminotransferase, mitochondrial	P29758	0.048	reduced
Moesin	P26041	0.007	reduced
Microtubule-associated protein 6	Q7TSJ2	0.008	reduced
Catenin (Cadherin associated protein), alpha 1	Q6NV50	0.043	reduced
N(G),N(G)-dimethylarginine dimethylaminohydrolase 1	Q9CWS0	0.009	reduced
Sodium- and chloride-dependent GABA transporter 3	P31650	0.037	reduced
UMP-CMP kinase	Q9DBP5	0.008	reduced
Catenin beta-1	Q02248	0.009	reduced
Vesicle-associated membrane protein-associated protein A	Q9WV55	0.030	reduced
Protein disulfide-isomerase A4	P08003	0.044	reduced
Importin subunit beta-1	P70168	0.029	reduced

**Table 3-:** Proteins differentially expressed in both NMDA and ONC groups

Protein Name	Accession Number	p-value control vs NMDA	p-value control vs ONC	Level- NMDA vs control	Level- ONC vs control
Ras-related protein Rab-18	P35293	0.002	0.002	reduced	reduced
DNA-(apurinic or apyrimidinic site) lyase	P28352	0.001	0.004	elevated	elevated
AP-1 complex subunit beta-1	O35643	0.024	0.005	reduced	reduced
UMP-CMP kinase	Q9DBP5	0.043	0.008	reduced	reduced
Microtubule-associated protein 6	Q7TSJ2	0.010	0.008	reduced	reduced
N(G),N(G)-dimethylarginine dimethylaminohydrolase 1	Q9CWS0	0.044	0.009	reduced	reduced
Catenin beta-1	Q02248	0.010	0.009	reduced	reduced
Eukaryotic initiation factor 4A-II	E9Q561	0.011	0.012	elevated	elevated
Small nuclear ribonucleoprotein D3	Q91VM2	0.031	0.013	elevated	reduced
Clathrin coat assembly protein AP180	Q61548	0.021	0.026	reduced	reduced
ATP synthase subunit d, mitochondrial	Q9DCX2	0.020	0.028	reduced	reduced
CST complex subunit STN1	Q8K2X3	0.049	0.028	elevated	elevated
Importin subunit beta-1	P70168	0.046	0.029	reduced	reduced
Heterogeneous nuclear ribonucleoprotein F	Q9Z2X1	0.018	0.029	elevated	elevated
Sodium- and chloride-dependent GABA transporter 3	P31650	0.044	0.037	reduced	reduced
Transforming protein RhoA	Q9QUI0	0.019	0.037	reduced	reduced
Fumarate hydratase, mitochondrial	P97807	0.009	0.038	reduced	reduced
Transportin-1	Q8BFY9	0.043	0.040	reduced	reduced
Catenin (Cadherin associated protein), alpha 1	Q6NV50	0.041	0.043	reduced	reduced
Carbonic anhydrase 14	Q9WVT6	0.012	0.045	reduced	reduced
DnaJ homolog subfamily C member 8	Q6NZB0	0.041	0.047	elevated	elevated
Ornithine aminotransferase, mitochondrial	P29758	0.047	0.048	reduced	reduced
